# Response to Comment on “Health outcomes and female genital mutilation/cutting: how much is due to the cutting itself?”

**DOI:** 10.1038/s41443-023-00670-z

**Published:** 2023-03-09

**Authors:** Sophia D. Koukoui, Georgia J. Michlig, Crista E. Johnson-Agbakwu

**Affiliations:** 1 Université de Montréal, Psychology Department, Montreal, Quebec Canada; 2 CIUSS Centre Ouest-de-l’ile-de-Montréal-Sherpa, Montreal, Quebec Canada; 3 Johns Hopkins Bloomberg School of Public Health, Baltimore, MD USA; 4 Southwest Interdisciplinary Research Center, Watts College of Public Service and Community Solutions, Arizona State University, Phoenix, AZ USA; 5 Refugee Women’s Health Clinic, Valleywise Health, Phoenix, AZ USA; 6 Creighton University School of Medicine, Phoenix Regional Campus, Phoenix, AZ USA; 7 District Medical Group, Phoenix, AZ USA

**Keywords:** Health sciences, Medical research, Risk factors

In her comment to our article, ‘Health outcomes and female genital mutilation/cutting: how much is due to the cutting itself?’ [1], the author stresses the need for scientific rigor in investigating the potential adverse health outcomes of FGM/C; a need for rigor which no scholar will dispute. The author offers a summary of common methodological shortcomings in the investigation of a link between FGM/C and adverse health outcomes, and factual physiology information to debunk erroneous statements related to FGM/C and obstetric complications.

With regards to our publication, the author’s main claim is that we drew conclusions beyond the scope of what our methodology allows for, and states that “there would still need to be more direct evidence of a mechanism linking those data to specific adverse outcomes among women with (or without) FGM/C before it would be appropriate to infer the suggested relationship of discrimination driving the adverse outcomes” [2]. This assertion suggests that the author has missed the mark with regards to the main contribution of our research. While we as well, put into question an exclusive focus on FGM/C as the sole contributor to potential adverse health outcome, the overarching goal of our research was to introduce a social lens that needs to be layered on top of both causality and biology.

The author puts into question the fact that we “did not ask any questions related to perceived discrimination or other activities occurring during health care encounters”. Given the abundant literature to that effect dating back to well over two decades, including our own work based on the same data set [1, 3] and our qualitative work on this issue [4], considering that the dire need for enhanced healthcare for women with FGM/C has already been acknowledged on a global scale, we purposely shifted the focus onto a novel perspective, most specifically: on the social forces that undergird these women’s daily lives and their potential health impact.

Several points should be highlighted here:

1. The author asks a series of pragmatic questions to illustrate the need to clarify the link between discrimination and adverse health outcome (e.g. “How does receiving poor service when visiting a restaurant or store, for example, relate to difficulties in getting pregnant or the occurrence of a genital tract infection?”). Given the timelines of research and its vicissitudes, the exact underlying mechanisms will not be identified tomorrow. Undoubtedly, a number of studies will be required to delineate the underlying mechanisms through which everyday discrimination impacts health outcomes. However, as scientists, we cannot ethically wait until underlying mechanisms are elucidated to draw attention to the adverse health effects of discrimination, even more so for an already marginalized population. Moreover, even an in-depth exegesis of the underlying mechanisms does not equate healthcare institutions’ structural and cultural competence [5].
2. The author’s recommendation to delve into the “mechanism linking those data to specific adverse outcomes”, would still be an incomplete analysis. Indeed, the differential impact of specific stress factors should be considered longitudinally. For example, it is possible that for a young girl who was recently cut, FGM/C is the greatest stress factor or determinant of health. Yet other factors may have a more adverse effect at other stages in her life. Indeed, there is much scientific value in thinking dynamically, developmentally, to consider time, context, network, and space (among other factors) in the conceptualisation of determinants and underlying mechanisms to adverse health outcomes. We encourage fluidity: to think outside the box and in this case, ‘outside the clinical space’, and to then iteratively explore further the reverberations, links and bi-directionality of clinical and social encounters.
3. Our data was collected at a time of great angst for the US-based Somali community (the ‘Muslim ban’). As addressed in our discussion section, studies conducted during the same period have highlighted the ensuing adverse psychosocial, community, and public health outcomes. As Young aptly states “both xenophobic rhetoric and legal maneuverings have ostracized many immigrant groups”, thereby constituting a public health challenge in health services and access for the Somali community in the US [6].
4. While geopolitical strains and the pervasive deleterious effects of discrimination on well-being continue to be unraveled mechanistically and through public health studies, we would like to highlight Kirmayer’s rich summary of scholarly research on the impact of othering on mental health from an international perspective and with a special emphasis on anti-Muslim rhetoric [7]. As well, while his work is not specific to FGM/C, the work of psychiatrist Yasser ad-Dab’bagh comes to mind, as he affords an explanatory psychological model on the intrapsychic impact of prejudice [8]. His follow-up work delves into large-group dynamics and discrimination in contemporary geopolitical environments, notably the United States [9].
5. While the impact of discrimination in healthcare on women’s health should undoubtedly be investigated, a crucial issue remains: the need for better metrics in assessing racism and discrimination in the healthcare system [10]. Several initiatives have been undertaken, notably the UCLA equity dashboards, and the PROMs standardized questionnaire to facilitate the identification of bias in treatment decision [11]. However, as aptly stated by Hamed et al., research on racism in healthcare is, at the present time, “mostly descriptive and atheoretical, uses racial categories uncritically and tends to ignore racialization processes making it difficult to conceptualize racism” [12]. Furthermore, most tools are developed among English-speakers, thus limiting its use by non-English speaking migrants, who notoriously face communication hurdles with healthcare providers.

It is only at the end of 2020 that racism was recognized as a public health crisis in the US. Yet, it impacts social determinants of health (such as employment, education, housing), which are key drivers of health inequity and poorer health outcomes. To the best of our knowledge, no other study to date has explored a potential link between the discrimination deeply woven into the very fabric of society and adverse health effects in women who have experienced FGM/C.

This study was not about discrimination in healthcare as driving negative health outcomes. It is about the sociopolitical environment in which these women are embedded; one which our data indicates negatively impacts their health, well beyond their FGM/C status. As crucial as quality healthcare is, factors outside the clinical encounter have a resonance on body and psyche.

We invite our colleagues to broaden the lens and forge a more global, multifactorial perspective as to the potential contributing factors to health outcomes of women with FGM/C.

**Fig. 1:**
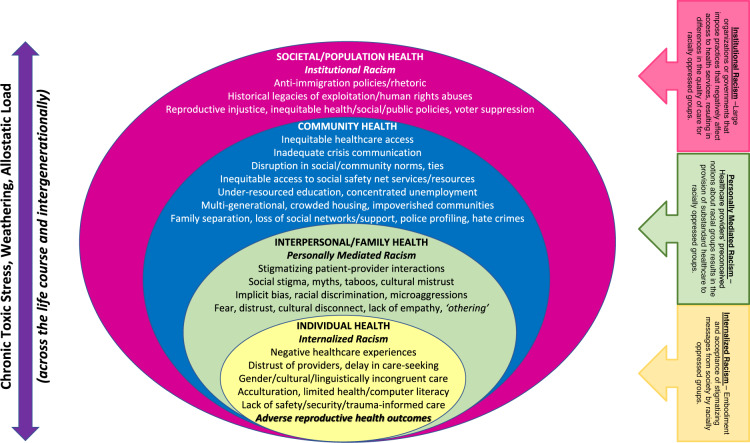
The Socio-Ecological Model of the Impact of Racism on the Sexual, Gender and Reproductive Health of BIPOC Women, Mothers and Birthing People, including migrant populations. Reprinted with Permission by Wolters Kluwer from: Johnson-Agbakwu [13].

Future critical scholarship on FGM/C must be inclusive of the larger socioecological framework within which women affected by FGM/C must navigate their health care Fig. 1.- ref. [13]. Women’s health care experiences cannot be divorced from the larger society within which they reside. Whether pushing upstream or examining downstream effects across the individual, interpersonal, community, societal, and global levels, there are myriad ways in which racism may exert a weathering effect through chronic toxic stress and allostatic load upon the lives of migrant women across their reproductive life course and intergenerationally, which manifests in the disproportionate Black maternal morbidity and mortality burden that is presently a public health and human rights crisis in the United States [14].

We invite FGM/C experts to consider the social and political constructions of the ‘Other’ and their impact on mental health and wellbeing; to interrogate the Healthy Migrant Paradox [13]; and to consider factors beyond race and ethnicity, to include ethnocultural granularity in examining one’s country of origin, length of time in the host nation, language, geospatial residential clustering, social support, and acculturative forces, among other factors.

In order to build upon this foundational scholarship, enhancing causal inference and methodological rigor, we invite future researchers engaging FGM/C-affected populations to design longitudinal community cohorts that consider a socioecological framework, employs mixed methods community-based participatory research (CBPR) approaches anchored in trust, and centers women with lived experience of FGM/C; incorporating validated and cross-culturally equivalent metrics of racism, bias, and discrimination.

Indeed, the hypothesis that discrimination has negative impacts on health is not a new one and is well documented in the literature. This includes the documented health impact of discrimination among immigrants [15]. Furthermore, that women affected by FGM/C experience discrimination beyond that related to their FGM/C status, and beyond the walls of the healthcare center, is documented in the qualitative literature [1].

When studying adverse health outcomes for women who have experienced FGM/C, neither FGM/C, nor racism, not even discrimination in healthcare “tells the whole story”. Nevertheless, no stone should be left unturned in our scholarly exploration of contributing factors, including the ones that can be as polarizing and uncomfortable to contemplate as social discrimination and immigration legislation. As the clinical space constitutes an echo chamber for historical and contemporary events, our reflection continues as to how our praxis and research methodologies account for the full breadth of our patients’ lived reality.
